# Learning in hybrid classes: the role of off-task activities

**DOI:** 10.1038/s41598-023-50962-z

**Published:** 2024-01-18

**Authors:** Carli Ochs, Caroline Gahrmann, Andreas Sonderegger

**Affiliations:** 1 Department of Psychology, Georgetown University, Washington, D.C. USA; 2 University of Fribourg, Fribourg, Switzerland; 3 Bern University of Applied Sciences, Bern, Switzerland

**Keywords:** Human behaviour, Psychology

## Abstract

Hybrid teaching (synchronous online and on-site teaching) offers many advantages (e.g., increased flexibility). However, previous research has suggested that students who join classes online suffer higher levels of distractibility, which might translate into students engaging in more off-task activities. This, in turn, can impair students’ learning performance. The following quasi-experimental field study investigated this specific link between teaching mode, engagement in off-task activities, and learning performance. We collected survey data from *N* = 690 students in six hybrid classes (*N* = 254 online, *N* = 436 on-site). Participants reported the amount of time they spent engaging in digital and non-digital off-task activities and responded to a quiz on the course material. Results revealed that online students spent more time engaging in off-task activities than on-site students. Further, results were consistent with our hypothesis that joining the class online is associated with lower learning performance via time spent on digital off-task activities.

## Introduction

Since the start of the SARS COVID-19 pandemic, hybrid teaching has risen to an unprecedented level. Hybrid teaching is a teaching format where students can synchronously follow the class on-site or online^1^. This was especially useful in the later stages of the pandemic because it allowed for non-vaccinated or immuno-compromised students to follow classes from the safety of their homes^2,3^.

Overall, hybrid classes have been argued to augment the accessibility of classes^1^. The flexibility of such a format allows people who live further away or have other commitments to follow the same lectures as students who can physically join the class^1,4^. The hybrid format is easy to set up: it often consists of a live stream of the on-site class with more or less opportunities for online students to interact (e.g., commenting using the chat function, unmuting the microphone and asking questions or answering questions of the teacher). Because of its reputed benefits and its low cost, there is a high probability that universities and other institutions in the education system will continue to employ hybrid teaching even once there is no longer need for social distancing.

However, a major concern with hybrid teaching is whether the quality of learning is the same for online and on-site students^5,6^. Previous research has argued that online students might face more distractions and consequently engage in more off-task activities, which may lead to lower learning performance^5,6^. Yet, little research has addressed this question empirically, and scientists have called for further research in this field^5,6^. Therefore, in the following study, we investigated student behavior and learning in hybrid teaching, specifically the effect of teaching mode (online vs. on-site) on learning performance mediated via off-task activities.

### Hybrid teaching: the difference between online and on-site learning

Hybrid classes allow online and on-site students to simultaneously follow the same class. However, while the class content is the same for both student groups, the learning environment is not. Online and on-site students’ environments may vary in terms of various physical, social and technological aspects, which might influence students’ learning engagement, experience of boredom, and distractions^7–10^. In the following paragraphs, we demonstrate how differences between the online and on-site environments may lead online students to be more vulnerable to distractions.

First, students following classes online might be in an overall more distracting environment. On-site students are in a classroom, (a supervised space dedicated to teaching and learning), while online students often are following their classes in a space that is cluttered, noisy and disturbing, hence less favorable to learning^11,12^.

Second, the social environment between both groups varies in terms of social control. On-site students are in a classroom where other students and the lecturer are physically present. This is not the case for online students. Indeed, students that were asked about their experience with online courses admitted that they miss the presence of peers and engage in more off-task activities because of a lack of social control^13^.

Third, computer-mediated communication may make it harder for online students to communicate with their teachers compared to on-site students^3,7^. For example, online students cannot discuss a matter face to face with a teacher after the course^7^. Furthermore, online students have reported feeling less comfortable asking questions due to slight delays caused by the online nature of the communication^3^. In addition, online students may face technical issues during class, such as internet connection and communication problems^7,14,15^. Both factors may lead online students to feel excluded or neglected^16^. Thus, students might feel disconnected from the class and, therefore, might be more vulnerable to distractions.

In summary, online students are likely to suffer from distraction due to an overall more distracting environment (cluttered, noisy and disturbing environment), reduced social control, and feelings of exclusion or neglect. All of these factors might impinge on students’ ability to concentrate and focus on learning content. Higher levels of distraction imply lower attention on the class.

### Impact of off-task activities on learning

Research has shown that students' attention may fluctuate during a lesson^6,17^ and that reduced attention may be associated with reduced learning performance^18–20^. This link has been established for various forms of attentional disengagement, such as mind wandering and engagement in digital as well as non-digital off-task activities^21–23^.

Mind wandering can be defined as “a situation in which executive control shifts away from a primary task to the processing of personal goals^24^”, p. 946. Mind wandering is prevalent in teaching and learning situations^12,25–27^. Various studies adopting different methodological approaches (e.g., using probes of attention in recorded video lectures or live courses, self-reports, diary studies etc.) have shown that the occurrence of mind wandering is negatively linked to academic performance^6,17,23,27,28^. Interestingly, more recent work has indicated that the negative effect of mind wandering has less of an impact compared to other indicators of attentional disengagement, such as engagement in digital off-task activities^29^.

Research on engagement in digital off-task activities (also referred to as multimedia multitasking) has primarily focused on different forms of digital off-task activities^22,30,31^, such as computer use in the classroom, e.g.,^32–34^, smartphone use in the classroom^19,35–40^, or the use of specific content such as social media^41–45^. Overall, empirical evidence mostly suggests that students regularly engage in digital off-task activities and that this negatively affects their learning^29,32,34,35,39^.

While the impact of digital off-task activities in the classroom have been studied quite intensely, there has been a relative lack of research on the impact of non-digital off-task activities^46^. One non-digital off-task activity that has received some attention is doodling. Doodling is defined as absentmindedly drawing symbols, figures, and patterns that are unrelated to the primary task (e.g., listening to the lecture). Results on doodling and learning performance are mixed. Doodling has been shown to positively influence the retention of information in an auditory task^47–49^ but to negatively influence retention and learning in visual tasks^21,50^. There is only little empirical research addressing the consequence of other types of non-digital off-task activities. This could be related to the fact that the range of such activities is very broad and diverse. Moreover, specific non-digital off-task activities, such as ironing clothing, cleaning, cooking, and yoga, can only be carried out when participating in the lesson online. This last type of attentional disengagement might be particularly relevant in hybrid lessons as online students have a wider variability of potential non-digital off-task activities they could engage in (e.g., cleaning or taking a walk). In this regard, one experimental lab study addressing the effect of doing laundry while following a recorded online lecture has indicated negative consequences on learning^46^.

Concerning hybrid teaching, previous research on attentional disengagement in hybrid classes and their effect on performance is scarce, and researchers have called for further research in this field^5,6^. One study on attentional disengagement and learning in the hybrid context examined how mind wandering affected learning performance between students watching a class live on-site versus watching a video of the class in a lab^6^. This study found that mind wandering decreased in the on-site class condition but increased in the lab condition. Students in the video-lecture showed non-significantly higher learning scores than in the on-site session. The authors suspected this was due to the students' sterile settings while they viewed the lesson (e.g., in a lab without phones or computers). Contrary to natural settings, students in the classroom were exposed to more digital distractions than those watching the lesson online. This may have confounded the results, as digital distractions also lead to attentional disengagement and have been linked to lower learning performance^35,40^.

In summary, mind wandering, and digital and non-digital off-task activities reflect attentional disengagement that can hinder learning. As mentioned in the preceding section, online students may be more distracted due to their learning environment and the lack of social control. There is little empirical evidence comparing digital and non-digital off-task activities in on-site and online classes and their effects on learning performance. Thus, we intend to fill this research gap by studying hybrid teaching and learning performance mediated by students' digital and non-digital off-task activities.

### The present study

In the present quasi-experimental mixed-method field study, we investigated the mediating effect of attentional disengagement between teaching mode (online vs. on-site) and learning performance. Specifically, we assessed the digital and non-digital activities of students as well as their learning performance in classes that were taught in hybrid form (i.e., students following the same class either on-site in a lecture theatre or online in a location of their choice). We were particularly interested in these two forms of attentional disengagement for the following reasons. First, digital off-task activities have been shown to be worse for learning than mind wandering. Second, non-digital activities are of particular interest in hybrid classes as the possibility of activities for online students is vast.

**Figure 1:**
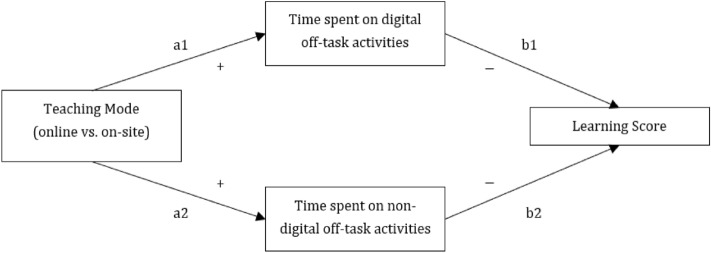
Proposed conceptual model.

Based on our review of the literature, we put forward the following hypotheses (a depiction of our proposed model can be found in Fig. 1):

*H1:* Teaching Mode is associated with time spent on digital off-task activities. Specifically, we expect students who participate online to spend more time on digital off-task activities than students who participate on-site.

*H2:* Teaching Mode is associated with time spent on non-digital off-task activities. Specifically, we expect students who participate online to spend more time on non-digital activities than students who participate on-site.

*H3:* Time spent on digital off-task activities mediates the relationship between teaching mode and learning score, such that online students spend more time on digital off-task activities, which, in turn, leads to a decreased learning score.

*H4:* Time spent on non-digital off-task activities mediates the relationship between teaching mode and learning score, such that online students spend more time on non-digital off-task activities, which, in turn, leads to a decreased learning score.

Teaching mode was coded in the following manner: 0 = on-site; 1 = online.

## Method

### Participants

We collected 690 responses to our survey (556 participants identified themselves as female, 129 as male, and 5 as other). The ages of participants ranged between 18 and 64 (*M* = 21.46; *SD* = 4.42). Participants were recruited at the end of a ninety-minute course in six different psychology lectures (i.e., introduction to work and organizational psychology (*N* = 47), clinical psychology (*N* = 107), introduction to developmental psychology (*N* = 161), introduction to cognitive psychology (*N* = 188), research methods (*N* = 142), diagnostical tests (*N* = 45) held in hybrid teaching mode by six different teachers. For each course some participants (total *N* = 436) were following a class on-site, and some participants (total *N* = 254) were following the class online. The choice whether to participate online or on-site was at the student’s discretion. In total, 74% of the students from these classes took part in the survey. Detailed information on participation rates per class and teaching mode can be found in the supplementary materials (Table S1). Based on Schönbrodt and Perugini (2013), we consider the total sample size of 690 as large enough to generate stable correlation estimates and offer a solid foundation for the correlation based statistical approaches used.

Observations of two independent raters indicated that the lectures did not differ considerably with regard to number of questions asked by the teacher (*M* = 7.33, *SD* = 6.57), online student interactions (*M* = 0.33, *SD* = 0.82), on-site student interactions (*M* = 7.33, *SD* = 5.49), technical problems (*M* = 0.25, *SD* = 0.61) and break duration (*M* = 10.83, *SD* = 5.15).

### Ethical approval

This study and its experimental protocols were approved by the Internal Review Board of the Department of Psychology at the University of Fribourg. All methods were carried out in accordance with relevant guidelines and regulations. Informed consent was obtained from all participants.

### Self-report measures

#### Questions regarding digital off-task activities

Participants were asked a series of questions about their computer/tablet and smartphone use during the lecture. These questions can be found in Table 1. Second-order questions (e.g., 1a. How many notifications/messages did you receive on your computer or tablet during this lecture?) were only presented if participants answered yes to the first-order question (e.g., 1. Did you use your computer or tablet during this lecture?). Items 2.a, and 3.c left enough space to report up to 5 activities.

**Table 1:** Questions regarding participants’ digital off-task activities during the lecture.

Computer use		
1	Did you use your computer/tablet during this lecture?	Yes*/No
1.a	How many notifications/messages did you receive on your computer/tablet during this lecture?	
2	Have you used your computer/tablet for activities other than those related to today's lecture?	Yes*/No
2.a	Which off-task activities did you do on your computer/tablet? Indicate the different activities and the estimated time you spent on doing them	
	Activity: ____________________	Estimated time: _____________________
Smartphone use		
3	Did you use your smartphone during this lecture?	Yes*/No
3.a	How many times did you pick up your smartphone or clicked the Main/Home button?	
3.b	How many notifications did you receive during this lecture?	
3.c	Which off-task activities did you do on your smartphone? Indicate the different activities and the estimated time you spent on doing them	
	Activity: ____________________ Estimated time: _____________________	

#### Questions regarding non-digital off-task activities

Participants were asked if they engaged in any off-task activity that was not performed on a digital device. If they did engage in such activities, they were asked to list the activities and estimate how much time they spent on each separate activity (cf. Table 2).

**Table 2:** Questions regarding participants’ non-digital off-task activities during the lecture.

1	Apart from the activities you have done on your digital devices, have you done any other activities that are not related to the course?	Yes*/No
1.a	Indicate the different activities and the estimated time you spent on doing them	
	Activity: ____________________ Estimated time: _____________________	

#### Context variables

To get a better understanding for the physical and mental context of participants, participants were asked to rate their experience on 5 items on a 7-point Likert scale (1 = totally disagree, 7 = totally agree) measuring their motivation (*During the class I felt motivated*), boredom (*During the class I felt bored.*), fatigue (*During the class I felt tired.*) as well as how engaged they perceived their teacher (*The teacher kept the subject interesting*.) and how distracting they rated their environment (*During this class my environment was distracting*.).

#### Learning score

Six multiple-choice questions on the content of the specific topic of the class were asked to assess students' learning scores. For each question, four answer options were provided, of which either one or two were correct. An example of a question can be found in Table 3. The learning score was based on the number of correctly responded answer options. Each question had 4 answer options, allowing participants to score 4 points per question. Therefore, the maximum achievable score is 24 (based on the multiplication of the number of question (6) and the number of answer options (4)).

**Table 3:** Exam example question.

1) Short term memory holds information for	
a	5 to 10 s
b	10 to 15 s
c	15 to 20 s
d	20 to 25 s

### Procedure

We implemented the exact same procedure for the six classes from which we recruited participants. Before each class, lecturers were contacted and asked for permission to perform recruitment in their class. Once they had agreed, we collaborated with them to create six exam questions on the course material for the upcoming class session. In each class, two researchers attended the course both online and on-site. Researchers sat at the back of the classroom and connected to the digital collaboration platform used to stream the class online (Microsoft Teams), where they listened with one earphone to the online class. With the other ear, they listened to the on-site class. This allowed them to code for aspects such as interactions on-site (questions from students) as well as interactions online (e.g., in chat) or issues with connections or lag.

The classes ended approximately 15 min before the scheduled time. The two researchers came to the front of the class and asked students to participate in the study. Students were told that the survey was on the effects of teaching mode (online/ on-site) on their learning. As incentives, participants were told that there would be a quiz on course material that might help them to prepare for the end-of-term exam, and participants were also rewarded points toward course credits. Great emphasis was given (orally and in written form on the first page of the survey) to the anonymous nature of the study and it was stressed that students should answer as honestly as possible. After the participants had confirmed their informed consent, they answered the questionnaire and responded to the quiz on the course material.

### Qualitative data analysis

Participants' responses regarding their digital and non-digital off-task activities were analyzed using inductive coding. Eleven categories were identified for digital off-task activities, and eleven were identified for non-digital off-task activities (category descriptions can be found in supplementary materials Table S2.). Activities were then coded into their respective categories by two coders. The inter-rater reliability was estimated using Cohen's kappa and showed to be satisfactory for digital off-task activities (*k* = 0.93) as well as for non-digital off-task activities (*k* = 0.97). The few differences in coding were then solved through discussion.

### Quantitative data analysis

We performed a single-level mediated linear regression analysis via the R package lavaan^51^ to probe our hypotheses. To ensure robust statistical inference while accounting for the nested structure of the data, we treated classrooms as fixed effects. This approach effectively addresses the issue of violating independence assumptions within clusters, ensuring the accuracy of our results without the inherent unreliability that would have ensued from a multilevel model with a small number of units at Level 2. Moreover, to account for the different learning tests employed across classes, we centered students' learning scores around their respective class mean.

Preceding the analysis, we checked for potential violations of relevant inference assumptions. We performed visual inspections of residuals. Our inspections did not reveal any apparent violations of the linearity assumption. However, residuals were non-normally distributed, thus violating the homoscedasticity assumption. We hence based our inference on bootstrapped confidence intervals (1.000 bootstrapped samples) to ensure robust estimation.

## Results

### Descriptive data

Table 4 presents a comprehensive overview of participant enrollment, delineated by class and learning environment, alongside their corresponding average learning scores. The data discerns that, on the whole, the mean learning scores per class tended to be higher for students attending on-site, with the exception of one class, where scores were higher for online participants.

**Table 4:** Selected descriptive information per classroom.

	*N* (%)		Learning score *M* (*SD*)	
	Online	On-site	Online	On-site
Classroom 1	68 (36.17)	120 (63.83)	18.56 (1.88)	18.80 (2.13)
Classroom 2	23 (48.94)	24 (51.06)	15.95 (2.40)	16.79 (2.21)
Classroom 3	17 (48.57)	28 (51.43)	17.18 (2.74)	18.46 (1.99)
Classroom 4	44 (30.99)	98 (69.01)	17.25 (2.74)	17.73 (2.39)
Classroom 5	71 (44.10)	90 (55.90)	18.69 (2.32)	18.56 (2.56)
Classroom 6	31 (29.00)	76 (71.03)	18.35 (2.33)	18.49 (2.50)

Table 5 reveals that a higher proportion of online students engaged in non-digital and digital activities compared to students that joined the class on-site. On average, online students also spent more time engaging in these activities. A table with the detailed summary of students’ percentage of activity and average time per specific activity can be found in supplementary materials (Table S3).

**Table 5:** Summary of students overall off-task activity.

	Online (N = 254)		On-site (N = 436)		Total (N = 690)	
	% of students that engaged in	Avg. time in min. (SD) on	% of students that engaged in	Avg time in min. (SD) on	% of students that engaged in	Avg time in min. (SD) on
Non-digital activities	46.06%	9.17 (12.4)	27.29%	6.62 (13.5)	34.20%	7.88 (12.98)
Digital activities	77.56%	16.21 (17.72)	64.91%	13.52 (19.98)	69.57%	14.61 (19.02)

Percentages were calculated based on the total number of participants in the respective groups.

Avg. time was calculated based on the time spent by students who engaged in the respective off-task activity. Thus, students who spent zero minutes on the respective off-task activity were dropped from this part of the analysis.

### Hypotheses testing

Table 6 shows the means, standard deviations, intra-class coefficients and intercorrelations of the study variables. All hypotheses were tested by means of a single level mediated linear regression model. Table 7 displays the results of this analysis.

**Table 6:** Means, standard deviations, intra-class coefficients and correlations among study variable.

	Variable	M	SD	ICC	1	2	3	4
1	Teaching Mode^a^				–			
2	Time^b^ on digital off-task activities	10.16	17.23	0.19	0.11*	–		
3	Time^b^ on non-digital off-task activities	2.70	8.45	0.00	0.14**	0.04	–	
4	Learning Scores	18.21	2.42	0.11	− 0.06	− 0.14**	− 0.02	–

N = 690.

^a^Coding of Teaching Mode: 0 = on-site; 1 = online, ^b^Time is indicated in minutes.

*p < 0.05. **p < 0.01. Test of direct effects.

**Table 7:** Summary of direct and indirect effects.

Effect	beta estimate	95% CI for beta estimate
Direct effects		
Teaching Mode^a^ →Time^b^ on digital off-task activities (a1)	3.76	[0.99; 6.42]
Teaching Mode^a^ → Time^b^ on non-digital off-task activities (a2)	2.40	[0.98; 3.75]
Time^b^ on digital off-task activities → learning success (b1)	− 0.02	[− 0.03; − 0.01]
Time^b^ on non-digital off-task activities → learning success (b2)	− 0.01	[− 0.03; 0.01]
Indirect effects		
Teaching Mode^a^ → Time^b^ on digital off-task activities → Learning Scores (a1b1)	− 0.07	[− 0.12; − 0.02]
Teaching Mode^a^ → Time^b^ on non-digital off-task activities → Learning Scores (a1b1)	− 0.02	[− 0.09; 0.01]
Total effect		
Teaching Mode^a^ → Time^b^ on digital off-task activities + Time^b^ on non-digital off-task activities → Learning Scores (a1b1 + a2b2)	− 0.09	[− 0.17; − 0.03]

*N* = 690 students.

Learning scores were centered around the respective class mean.

*R*^*2*^ = 0.06 for student learning success.

^a^0 = on-site; 1 = online. ^b^Time is indicated in minutes.

We proposed a direct association between teaching mode and time spent on digital (H1) and non-digital (H2) off-task activities by students in hybrid classrooms. In line with H1, data analysis (see Table 7) revealed that teaching mode was associated with time spent on digital off-task activities, with students who participated online spending more time on digital off-task activities than students who participated on-site (*a1* = 3.76, *95%* CI [0.99; 6.42]). Hypothesis 1 was thus supported. In line with H2, teaching mode was associated with time spent on non-digital off-task activities, with students who participated online spending more time on non-digital off-task activities than students who participated on-site (*a2* = 2.40, *95%* CI [0.98; 3.75]). Hypothesis 2 was thus supported.

#### Test of indirect effects

We estimated the total effect of teaching mode on students' learning performance (*a1b1* + *a2b2* =  − 0.09, *95%* CI [− 0.17; − 0.03]). This suggests that, on the learning performance scale ranging from 0 to 24 points, the expected difference in learning performance between a student attending online and one attending on-site is 0.09 points, with the online student being expected to score lower. Crucially, this total effect is attributed entirely to the indirect influences of teaching mode on learning outcomes through engagement in both digital and non-digital off-task activities. Specifically, we assumed time spent on digital (H3) and non-digital (H4) off-task activities to mediate the relationship between teaching mode and learning scores. Our results revealed an indirect effect of teaching mode via time spent on digital off-task activities on learning scores (*a1b1* =  − 0.07, *95%* CI [− 0.12; − 0.02]), nominally accounting for 77.78% of the total effect of teaching mode on learning performance detailed above. Hypothesis 3 was thus supported. However, our results did not reveal an indirect effect of teaching mode via time spent on non-digital off-task activities on learning scores (*a2b2* =  − 0.02, *95%* CI [− 0.09; 0.01]), nominally accounting for only 22.22% of the total effect of teaching mode on learning performance detailed above. Hypothesis 4 was thus not supported.

### Analysis of context variables

The analysis of the context variables revealed very small, non-significant differences between online and on-site ratings of student motivation, experienced boredom and fatigue, as well as the evaluation of the teacher’s engagement. The environment however was rated as more distracting by students taking the class online compared to students participating on-site. A table summarizing the results can be found in the supplementary materials (see Table S4).

## Discussion

This study aimed to evaluate potential differences in levels of distractibility and learning performance between online and on-site students in hybrid classrooms. As predicted, we found that students following a class online spend more time on digital and non-digital off-task activities than on-site students. Results were consistent with our hypothesis that engagement in digital off-task activities mediates a detrimental effect of online teaching on learning. Notably, results were not consistent with our hypothesis that engagement in non-digital off-task activities mediates a detrimental effect of online teaching on learning.

The results regarding the effects of teaching mode on engagement in off-task activities corroborate the assumptions outlined in the introduction. The descriptive data revealed that online students did not only engage in more off-task activities overall, but they also engaged in these activities for longer compared to on-site students. Some of the off-task activities were exclusively carried out by online students. This might be because some activities can only be carried out at home (e.g., cat care) while others might be considered inappropriate in class (e.g., watching a video). These examples indicate that online students might be confronted with more potential distractors and might feel less restricted to engage in certain behaviors due to a lack of social control.

This pattern of results indicates that despite the important advantages of hybrid teaching (e.g., increased accessibility of teaching), online students are particularly vulnerable within this setting: They report a higher degree of distractibility that is linked with an increased engagement in off-task activities. It is important to note that the teachers participating in this study did not implement any measures to optimize online students' learning experiences such as regularly integrating online students into class discussions, or using online quizzes and other tools to generate an interactive learning environment^1,52,53^. The focus on on-site students and the absence of particular measures to improve online students’ experience observed in this study may be attributable to the particular context of the COVID-19 pandemic. These classes were held as a temporary solution, meaning that they were not originally conceived as hybrid classes and that teachers had not undergone training on how to ideally teach in such a format^54^. Therefore, future research should address this research question in courses that are more geared towards integrating online students. Investigating hybrid teaching during the pandemic is important since the experiences made during this period are bound to influence and shape future use and understanding of hybrid teaching^15,54–56^. For example, now that teachers and institutions have discovered how simple it is to live stream a class, they might do this more frequently without investigating any further into the potential pitfalls of this format.

Regarding the effect of off-task activities on learning, the question arises why students' engagement in non-digital off-task activities did not show the same negative effects as the engagement in digital off-task activities. This might be related to the nature of the activities students engaged in. Most of the reported non-digital activities are manual tasks (e.g., eating and drinking, drawing), while digital activities mainly require verbal and visual resources (e.g., writing text messages and reading). Listening to a class and learning are tasks that require verbal resources^46^. Wickens’ Multiple Resource Theory^57^ states that individuals can engage concurrently in tasks that require different resources, such as verbal and manual resources, without negative consequences on task performance. In contrast, concurrent engagement in tasks that require similar resources is linked to reduced task performance. In the context of learning, such effects have been shown already in research on doodling, where positive effects of absentminded drawing (a task requiring some visual resources) on learning were reported for auditory tasks^47–49^, while negative effects were observed for visual tasks^21,50^. In this study, verbal non-digital off-task activities (e.g., social interactions or writing and reading) were less common than manual ones. In contrast, reported digital activities relied largely on students' verbal and visual resources, interfering with class support.

Our findings have several practical and scientific implications. First, we found that student, especially online students, in hybrid lessons engage in off-task activities. This shows the need of interactive and engaging teaching to keep students focused on class topic and away from off-task activities. Online students should be reminded to study in a distraction-free atmosphere because they are more susceptible to distraction. Second, this study also revealed a negative effect of digital off-task activities on learning performance. Therefore, it is important to consider measures such as warning students of the negative consequences of partaking in off-task activities as well as suggesting rules of conduct regarding digital devices during class. Third, concerning non-digital off-task activities, a negative effect on learning was not found as, most likely, most activities did not interfere with the resources required for learning. However, for future research it might be interesting to conduct a more fine-grained analysis of the specific off-task activities. Lastly, while literature on how to conduct good hybrid teaching was available, it was not used in the examined classes. This suggests a gap between practice and research and should stimulate reflection on how to effectively communicate this knowledge to practitioners.

There are limitations to the present study. First, due to the quasi-experimental nature of the study design, participants self-selected their teaching mode (online vs. on-site). A random assignment of students to the two teaching modes was not possible because of ethical and legal constraints. In other studies, this problem was circumvented by simulating a course in the laboratory^6,17^. However, this implies that the occurrence of off-task activities cannot be observed in a naturalistic environment, which severely undermines the ecological validity of any study. Therefore, we are convinced that this first limitation regarding randomization is outweighed by gains in ecological validity. Second, the assessment of participants' engagement and time spent on off-task activities was based on self-reports. This might be problematic for two reasons: (1) a social desirability bias, (2) memory distortions. To reduce the risk of (1) a social desirability bias, we highlighted the importance of answering the survey honestly and correctly and stressed the anonymity of the data. With regard to potential (2) memory distortions, results of a meta-analysis indicated that self-report data only moderately correlated with objective measures of technology use^58^. This meta-analysis, however, summarized data from studies comparing self-reports and objective technology use over multiple days and weeks, while this study recorded self-reports for the last 90 min. For such short time frames, previous research has indicated that self-report measures regarding technology use are reliable^59^.

Other, more objective ways to assess students’ off-task activities in class would imply installing tracking software on students’ devices or using thought-probe techniques, all of which would reveal the nature of the study to the students. Because previous research has shown that knowledge about the objective of a study can influence participants’ behavior during the study^60^, it was important to us that the participants have no previous knowledge about our research objectives. Other researchers have made similar methodological choices and used self-reports for their studies in this context as well^35,59,61,62^. Third, we only recruited participants in psychology classes. For generalization purposes it would be ideal to conduct such a study in classes of other fields of study. Lastly, we note that teaching mode and off-task activities explained a relatively small portion of variance in students' learning scores overall. While learning performance represents a particularly important objective teaching outcome, it is endogenous to many contextual and student-specific factors^63^. Considering any one factor is therefore bound to explain relatively limited proportions of variance.

## Conclusion

In conclusion, while hybrid teaching might be a useful tool to make classes more accessible, it does not guarantee equal opportunities in terms of learning performance. To ensure that students have an equal opportunity to achieve important learning objectives, instructors must be aware of the negative consequences of off-task activities, especially digital off-task activities for students participating online. In addition, they must adapt to the specific requirements of hybrid teaching in order to create optimal learning conditions for both on-site and online students. At the same time, students need to be made aware of creating remote learning conditions that offer as few distractions as possible and enable concentrated and focused learning.

Taking these measures into account is extremely important. Indeed, the past pandemic has shown that individuals with special needs, such as those who are immunocompromised or have physical disabilities, and those with family obligations benefit from being able to participate in courses online. Therefore, it is an ethical and social necessity to create learning environments that do not disadvantage them and enable them to succeed in their studies.

### Supplementary Information

Supplementary Information.

## Data Availability

The dataset is available on OSF (https://osf.io/q4vf5/?view_only=21662f508cf44f68be78794d55f83be2).
