# Supplementary material for: Learning in hybrid classes: the role of off-task activities
Source: Sci Rep. 2024 Jan 18;14:1629. doi: 10.1038/s41598-023-50962-z (PMC10796355; doi:10.1038/s41598-023-50962-z)
Supplement: Supplementary file 1 — Supplementary Information. [file 41598_2023_50962_MOESM1_ESM.docx]

Table S1. Participation rate per class and teaching mode

|  | N Students | | N Participants | | % of participation | |
| --- | --- | --- | --- | --- | --- | --- |
|  | On-site | Online | On-site | Online | On-site | Online |
| Introduction to cognitive psychology | 128 | 88 | 120 | 68 | 93.75 | 77.27 |
| Organizational psychology | 26 | 26 | 24 | 23 | 92.31 | 88.46 |
| Diagnostical tests | 34 | 32 | 28 | 17 | 82.35 | 53.13 |
| Research methods | 110 | 80 | 98 | 44 | 89.09 | 55 |
| Introduction to developmental psychology | 107 | 108 | 90 | 71 | 84.11 | 65.74 |
| Clinical psychology | 135 | 55 | 76 | 31 | 56.30 | 56.36 |

Table S2. Description of types of non-digital and digital activities

| **Non-digital activities** | Definition | **Digital activities** | Definition |
| --- | --- | --- | --- |
| **Bathroom** | Going to the restroom. | **Activities other class** | Activities related to classwork from other classes. |
| **Cat care** | Activities involving cats (e.g., opening doors and petting) | **E-mail** | Sending or checking email. We chose to distinguish emails from instant messages as the format and time invested in their writing is typically different. |
| **Cleaning** | Cleaning or tidying up. | **Games** | Playing games such as solitaire. |
| **Daydreaming** | Looking out the window, staring into the distance and/or thinking of later events. | **Instant messaging** | Mention of instant messaging on any platform. |
| **Drawing** | Drawing and doodling | **Internet searches** | Any internet searches participants described. |
| **Eating and drinking** | Drinking water or eating snacks. | **News** | Reading the news |
| **Organizing/ planning** | Writing to do lists and looking in calendar. | **Online shopping** | Mention of looking up products or being on online shopping sites. |
| **Other** | Any other non-digital activity that did not fit in these categories but not appear enough to warrant its own category. | **Organization** | Activities related to the planning or organization of a future event (buying a train ticket, looking at their diary or calendar, or looking at a program). |
| **Selfcare** | Activites that are meant to do well to do body and mind such as putting on lotion but also stretching and doing sports. | **Other** | Any other digital activity that did not fit in these categories but not appear enough to warrant its own category. |
| **Social interactions** | Talking with friends and classmates. | **Social media** | Being on social networks such as Instagram, Facebook, and TikTok. Warning: if participants specify that they write messages, they are coded under instant messaging. |
| **Writing and reading** | Any form of writing and reading that that is not related to the class or conducted on a digital device. | **Video** | Watching any form of video (e.g., sports game, YouTube and TV show) |

Table S3. Detailed summary of students’ non-digital and digital off-task activities.

|  | Total (N=690) | | Online (N=254) | | On-site (N=435) | |
| --- | --- | --- | --- | --- | --- | --- |
|  | % of mentions | Avg time of use in min. (SD) | % of mentions | Avg time of use in min. (SD) | % of mentions | Avg time of use in min. (SD) |
| **Non-digital activities** |  |  |  |  |  |  |
| Eating and drinking | 35.56% | 3.41 (3.48) | 30.31% | 4.64 (4.06) | 17.89% | 2.2 (2.25) |
| Other | 4.49% | 12.69 (19.23) | 8.66% | 8.7 (10.01) | 2.06% | 22.43 (31.19) |
| Social interactions | 4.2% | 5.97 (5.53) | 5.12% | 6.08 (4.52) | 3.66% | 5.88 (6.38) |
| Drawing | 2.75% | 11.18 (13.95) | 3.54% | 13.94 (19.52) | 2.29% | 8.7 (5.98) |
| Daydreaming | 2.17% | 8.13 (5.33) | 2.76% | 8.71 (4.46) | 1.83% | 7.62 (6.25) |
| Selfcare | 2.03% | 3.44 (1.68) | 3.54% | 4.83 (4.81) | 1.15% | .82 (.74) |
| Organizing/ planning | 1.74% | 4.92 (5.5) | 2.36% | 8.25 (6.27) | 1.38% | 1.58 (.66) |
| Cleaning | 1.3% | 5.39 (6.01) | 3.15% | 6 (6.12) | 0.23% | 0.5 |
| Cat care | 1.16% | 3.44 (1.68) | 3.15% | 3.44 (1.68) | - | - |
| Writing and reading | 0.72% | 39 (33.43) | 1.18% | 18.33 (23.09) | 0.46% | 70 (14.14) |
| Bathroom | 0.72% | 2.8 (1.3) | 1.97% | 2.8 (1.3) | - | - |
| **Digital activities** |  |  |  |  |  |  |
| Instant messaging | 57.1% | 6.79 (7.66) | 64.17% | 6.75 (6.11) | 52.98% | 6.82 (8.6) |
| Social media | 19.42% | 6.96 (6.82) | 29.52% | 7.57 (7.04) | 13.53% | 6.19 (6.5) |
| E-mail | 13.48% | 3.66 (3.73) | 14.96% | 4.21 (4.87) | 12.61% | 3.28 (2.65) |
| Other | 10.29% | 7.69 (12.17) | 10.63% | 5.96 (7.76) | 10.09% | 8.75 (14.2) |
| Activities other class | 4.78% | 26.29 (21.68) | 3.94% | 30 (19.72) | 5.27% | 24.67 (22.7) |
| Internet searches | 4.9% | 6.71 (8.96) | 5.52% | 7.04 (11.33) | 4.36% | 6.47 (7.17) |
| Organization | 4.49% | 6.59 (12.01) | 4.72% | 4.33 (4.72) | 4.36% | 8.01 (14.88) |
| Games | 3% | 15.98 (14.82) | 6.3% | 16.66 (15.15) | 3.21% | 15.21 (14.96) |
| Online shopping | 2.75% | 9.05 (6.54) | 3.54% | 6.89 (6.25) | 2.29% | 11 (6.48) |
| News | 2.46% | 7.69 (12.17) | 1.57% | 7 (3.56) | 2.98% | 5.56 (5.26) |
| Video | 1.59% | 10.59 (8.84) | 4.33% | 10.59 (8.84) | - | - |

Percentage where calculated based on the total number of participants in the concerned groups

Table S4. Comparison of context variables as a function of teaching mode

|  | Online (N=254) | | On-site (N=436) | |  | | | |
| --- | --- | --- | --- | --- | --- | --- | --- | --- |
|  | M | SD | M | SD | t_Welch_ | df | *p* | *d* |
| Motivation | 4.7 | 1.4 | 4.9 | 1.4 | -1.54 | 537 | .123 | -0.12 |
| Boredom | 3.1 | 1.5 | 2.9 | 1.5 | 1.74 | 527 | .082 | 0.14 |
| Fatigue | 4.1 | 1.8 | 4.1 | 1.9 | 0.05 | 533 | .961 | 0.00 |
| Teacher engagement | 6.4 | 0.9 | 6.4 | 1.0 | 0.83 | 563 | .404 | 0.07 |
| Distracting environment | 3.6 | 1.9 | 2.9 | 1.6 | 4.66 | 453 | < .001 | 0.38 |
